# No Money, No Problem: Enhanced Reward Positivity in the Absence of Monetary Reward

**DOI:** 10.3389/fnhum.2019.00041

**Published:** 2019-02-12

**Authors:** Edward Tunison, Rourke Sylvain, Jamie Sterr, Vanessa Hiley, Joshua M. Carlson

**Affiliations:** Department of Psychological Science, Northern Michigan University, Marquette, MI, United States

**Keywords:** monetary, reward positivity, EEG, feedback negativity, medial frontal negativity, feedback error-related negativity

## Abstract

The reward-related positivity (RewP) is an event-related potential (ERP) with a positive amplitude occurring approximately 250–350 ms post-feedback at frontocentral electroencephalogram (EEG) electrode sites. The RewP is typically elicited in monetary gambling tasks and has a relatively larger amplitude for positive vs. negative outcomes. However, the extent to which RewP amplitude is modulated by non-monetary feedback is less clear. To address this issue, EEG was used to record reward-related electrocortical activity during a simple non-monetary gambling task. We hypothesized that the RewP would be enhanced for non-monetary wins relative to losses, which was supported by the results. In our supplementary material, we provide additional analyses suggesting that this effect was not observed for the P3. In sum, RewP amplitudes were larger for positive (nonmonetary) feedback relative to negative feedback at frontocentral electrode sites—suggesting that monetary reward is not necessary to elicit the RewP.

## Introduction

The reward-related positivity (RewP) is an event-related potential (ERP) with a positive amplitude occurring approximately 250–350 ms post-feedback at frontocentral electroencephalogram (EEG) electrode sites. The RewP is derived from earlier ERP terminology including the feedback negativity (FN), feedback-related negativity (FRN), medial frontal negativity (MFN), and feedback error-related negativity (FERN) used to describe this component (Miltner et al., 1997; Holroyd et al., 2008; Proudfit, 2015; Krigolson, 2017). However, RewP amplitude is relatively larger for rewarding or positive feedback compared to neutral or negative feedback and therefore more accurately reflects neural activity associated with reward (rather than error) processing (Holroyd et al., 2008; Proudfit, 2015). In particular, the RewP is thought to index reward-related activity in the mesocortical dopamine system (Holroyd and Coles, 2002; Holroyd et al., 2004).

In support of this point of view, converging evidence from functional and structural magnetic resonance imaging studies have linked RewP amplitude to activity in the ventral striatum and other reward system circuitry (Carlson et al., 2011; Becker et al., 2014), as well as the volume of dopaminergic midbrain structures such as the ventral tegmental area (Carlson et al., 2015). At a behavioral level, variability in RewP amplitude has been shown to correlate with symptoms of blunted reward processing in anhedonic depression (Foti et al., 2014; Liu et al., 2014) and serve as a risk factor for the development of major depressive disorder (Bress et al., 2013). Conversely, RewP amplitudes are elevated in individuals with higher levels of self-reported reward responsiveness (Bress and Hajcak, 2013) and is sensitive to reward magnitude (San Martín et al., 2010). Thus, RewP amplitudes can be thought of as a neural index of reward reactivity.

Most studies use simple gambling tasks with monetary rewards/wins and non-rewards/losses to elicit RewP responses (e.g., Hajcak et al., 2006; Holroyd et al., 2008; Proudfit, 2015). Recently, however, RewP amplitudes have been studied in other contexts. For example, one study found an enhanced RewP for monetary, but not social (i.e., a smiling face), rewards (Flores et al., 2015). In addition, a study by Weinberg et al. (2014) found that RewP amplitudes were elevated in conditions where participants received monetary rewards relative to conditions where participants received non-monetary feedback. This study also found that participants elicited a (non-significant) trend-level enhancement of the RewP for wins vs. losses in the non-monetary condition. Similarly, RewP amplitudes have been found to be enhanced for non-monetary points, but not rewarding images (Brown and Cavanagh, 2018). Thus, the RewP appears to be most sensitive to monetary feedback, but may also be sensitive to rewarding non-monetary feedback as well. This pattern of results is consistent with previous findings that feedback processing reflects the relative value of an outcome based on the range of possible outcomes rather than the objective outcome value (Holroyd et al., 2004). That is, the abovementioned studies have included multiple reward values (e.g., monetary and non-monetary) and in this context RewP amplitudes for non-monterary rewards may be relatively smaller due to the relatively lower value associated with non-monetary rewards compared to monetary rewards.

Given that the evidence for RewP reactivity to non-monetary rewards in gambling tasks is based on a limited number of studies that include of a range of possible reward outcomes, the sensitivity of the RewP to non-monetary feedback warrants further investigation. The primary aim of this study was to directly compare RewP amplitudes for wins and losses in a non-monetary (i.e., point-based) version of a simple gambling task with only two outcome types: wins and losses. We hypothesized that RewP amplitudes would be larger for non-monetary wins compared to losses.

## Materials and Methods

### Participants

Thirty undergraduate students (female = 21, right handed = 29) between the age of 18–26 (*M* = 19.7, *SD* = 2.05) provided informed consent and participated in the study for course credit. Data were excluded for four participants who did not have at least 20 artifact free trials in all conditions (e.g., Marco-Pallares et al., 2011) and an additional four participants due to technical problems (e.g., experiment crashed during testing session). The remaining sample consisted of 22 students (female = 16, right handed = 21, *M =* 19.45, *SD* = 1.74). The appropriate sample size was determined by utilizing the effect size reported in Weinberg et al. (2014) for the non-monetary win vs. loss difference. In particular, using G*Power 3.1.9.2 with *d* = 0.58, *α* = 0.05, and power = 0.80 it was determined that an *N* ≥ 20 would be needed to detect win > loss RewP amplitudes. The study was approved by the Northern Michigan University Human Subjects Committee. All participants provided informed written consent.

### Gambling “Doors” Task

The task was based on previous RewP studies using monetary rewards (Hajcak et al., 2006; Carlson et al., 2011), but modified to use non-monetary (i.e., point-based) rewards and losses. Points in the current study were entirely abstract and not exchanged for prizes at the end of the study. The task was programmed and administered using E-prime (Psychology Software Tools, Pittsburg, PA, USA) and trial events were linked to Net Station acquisition software (Phillips Neuro, Eugene, OR, USA).

**Figure 1:**
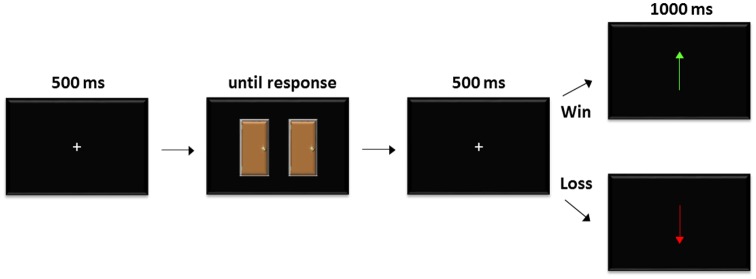
Participants were told to make a choice in door, and that one door contained a gain in points (win) and the other contained a decrease in points (loss). After a choice, predetermined feedback was given (120 wins and 120 losses).

As seen in Figure 1, each trial began with a white fixation cue (+) centered on a black background for 500 ms. Two doors were then simultaneously presented side-by-side on the horizontal midline. Participants were instructed that behind one door there was a gain of points and behind the other door there was a loss of points; they were then instructed to choose one door. The doors remained until a response was made. Afterwards, the fixation cue (+) re-appeared for 500 ms and was followed by feedback indicating a win/gain or loss of points. Gains (+1,250 points) were indicated by a green upwards arrow and losses (−625 points) were indicated by a red downwards arrow (losses perceived twice as valuable as wins; Tversky and Kahneman, 1992). Unknown to participants, feedback was pre-determined and randomized with 120 wins and 120 losses.

### EEG Acquisition and Data Processing

Continuous EEG recordings were collected using EGI’s HydroCel GSN 130 series 64-electrode cap based on a 10/20 system. The sampling rate for data digitization was 250 Hz. Similar to other studies using the EGI net, electrode impedances were kept under 75 kΩ (Carlson and Reinke, 2010; Rizer et al., 2018). All aspects of EEG data processing were performed with EGI NetStation Waveform Tools. Continuous EEG data were low-pass filtered at 30 Hz and high-pass filtered at 0.1 Hz. The EEG segmentation was time-locked to the stimulus feedback with 200 ms prior to feedback and 600 ms post feedback. Data then underwent an artifact detection process where amplitude deflections of at least 140 μV at eye-blink electrodes were considered eye-blinks and amplitude deflections of  55 μV or greater at eye-movement electrodes were considered horizontal eye-movements. Segments containing eye-blinks or eye movements were excluded from data analysis. Additionally, segments with more than 10 bad channels were discarded. Channels were considered bad in each segment if the fast average amplitude exceeded 200 μV (this is a weighted running average algorithm within the NetStation software where a single data point exceeding threshold would not necessarily be marked as a bad channel, but several beyond threshold data points would be marked as bad), the differential average amplitude exceeded 100 μV, or a channel displayed zero variance. Additionally, channels were considered bad and replaced across segments if they met the above-mentioned criteria in more than 20% of segments. Bad channels were replaced with interpolated data using spherical splines from the remaining channels. The ERP segments were then averaged for each participant so that each electrode had a single waveform for each condition. The averaged segments were re-referenced to the average of all electrodes. A baseline correction of −100 to 0 ms was applied. Based on visual inspection, average RewP amplitudes were extracted between 250–300 ms from electrode FCz and Cz (Proudfit, 2015) for each participant.

## Results

**Figure 2:**
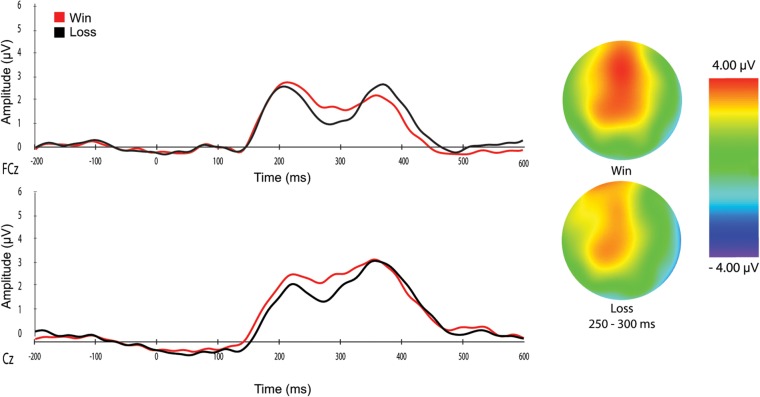
(Left) Reward-related positivity (RewP) was maximally elicited at frontocentral electrode cites FCz and Cz. Amplitudes for wins (red) were larger than losses (black). (Right) A map of electrocortical polarity at 250–300 ms after presentation of either win or loss feedback. Areas in red reflect maximal positive amplitudes.

A 2 × 2 repeated measures analysis of variance (ANOVA) was used to assess the effects of feedback type and electrode location on RewP amplitudes. There was an effect of feedback type, *F*_(1,21)_ = 8.90, *p* = 0.007, *η*_p_ = 0.30, where wins (*M* = 3.82 μV, *SE* = 0.56) resulted in larger RewP amplitudes compared to losses (*M* = 2.66 μV, *SE* = 0.42). As displayed in Figure 2, this was true at both electrode FCz (Wins: *M* = 3.08 μV, *SE* = 0.63; Losses: *M* = 2.05 μV, *SE* = 0.51, *p* = 0.01) and Cz (Wins: *M* = 4.56 μV, *SE* = 0.61; Losses: *M* = 3.26 μV, *SE* = 0.43, *p* = 0.01). There was a main effect of electrode location (*F*_(1,21)_ = 8.70, *p* = 0.008, $\eta_{\text{p}}^{2}$ = 0.29) where amplitudes were larger at Cz (*M* = 3.91 μV, *SE* = 0.47) relative to FCz (*M* = 2.56 μV, *SE* = 0.54); however, the feedback type × electrode location interaction was not significant, *F*_(1,21)_ = 0.58, *p* = 0.45, *η*_p_ = 0.03.

Given that the timeframe of the RewP overlaps with the P3, we preformed supplementary analyses on the P3. The P3 was not sensitive to outcome type (see Supplementary Material).

## Discussion

In this study, we used a well-defined gambling paradigm (Hajcak et al., 2006) to measure electrocortical responses associated with non-monetary gain or loss feedback. As hypothesized, we saw larger RewP amplitudes for non-monetary gains compared to non-monetary losses. The timing and location of this effect is consistent with previous research (Proudfit, 2015). As shown in Figure 2, the RewP was maximally observed at frontocentral electrodes (FCz and Cz) with a peak amplitude occurring approximately 280 ms post-feedback. The enhanced RewP for non-monetary wins observed in our results is consistent with previous findings that RewP amplitudes are modulated in non-monetary tasks (Weinberg et al., 2014; Brown and Cavanagh, 2018).

Given our results, in conjunction with previous studies (Weinberg et al., 2014; Brown and Cavanagh, 2018), it appears a financial incentive for participants may not be necessary. On the other hand, previous studies have shown that RewP (and other ERP) amplitudes are enhanced for monetized (relative to non-monetized) rewards (Van den Berg et al., 2012; Weinberg et al., 2014). Therefore, researchers will want to weigh the pros and cons of paying participants in their particular studies. Yet, for researchers wondering if it is *necessary* to pay participants to elicit the RewP, our results suggest the answer is no—the RewP can be measured without monetary incentive. This detail may be important for labs or institutions with limited financial resources.

Although we observed a RewP for non-monetary wins and losses in a simple gambling task, other forms of non-monetary rewards such as smiling faces (Flores et al., 2015) or other types of pleasant images (Brown and Cavanagh, 2018) do not appear to be effective in eliciting reward system activity as measured by the RewP. Previous research has shown that approach motivated states (Threadgill and Gable, 2016, 2018) or motivation for particular reward types (Angus et al., 2015) can affect RewP amplitude. Taken together, our findings and previous research (i.e., Weinberg et al., 2014; Flores et al., 2015; Brown and Cavanagh, 2018) indicate that non-monetary points or non-monetary wins in gambling tasks are more motivationally salient than other non-monetary reward types. This may be due to the fact that points are generally accumulated and can be used to gage one’s performance over time, whereas rewarding images are rewarding in themselves, but are not cumulative in nature. Given that point-based and other types of non-monetary feedback are ubiquitous in popular culture—e.g., likes on Facebook and other social media outlets as well as badges and points in online video games—it should not be surprising that non-monetary wins elicit reward system activity.

The RewP observed in the present study appears to mirror that seen in studies using monetary outcomes. However, we did not measure the RewP in a separate group receiving monetary feedback. Thus, from the current results we can only conclude that non-monetary positive feedback is rewarding and enhances RewP amplitude. The degree to which monetary and non-monetary RewP responses are one in the same will require further research.

## Author Contributions

JC designed the study and analyzed the data and significantly edited and revised the manuscript. ET, RS, JS, and VH collected data and drafted the manuscript.

## Conflict of Interest Statement

The authors declare that the research was conducted in the absence of any commercial or financial relationships that could be construed as a potential conflict of interest.
