# Supplementary material for: No Money, No Problem: Enhanced Reward Positivity in the Absence of Monetary Reward
Source: Front Hum Neurosci. 2019 Feb 12;13:41. doi: 10.3389/fnhum.2019.00041 (PMC6379288; doi:10.3389/fnhum.2019.00041)
Supplement: Supplementary file 1 [file Data_Sheet_1.docx]

**Supplementary Material**

*P3 Analysis*

Based on visual inspection, average P3 amplitudes were extracted between 300-350 ms post-feedback at electrodes FCz, Cz, and Pz for each participant. A 2 × 3 repeated measures analysis of variance (ANOVA) was used to assess the effects of feedback type and electrode location on P3 amplitudes. The Geenhouse-Geisser and Bonferroni corrections were applied when appropriate. There was no effect of feedback type, *F* (1, 42) = 0.72, *p* = .41, 𝜂_p_^2^ = .03. P3 amplitudes for wins (*M* = 3.87 μV, *SE* = 0.42) and losses (*M* = 3.60 μV, *SE* = 0.35) were approximately equal. There was a significant effect of electrode location (*F* (1, 42) = 6.41, *p* = .01, 𝜂_p_^2^ = .23) where amplitudes were larger at Cz (*M* = 5.20 μV, *SE* = 0.51) relative to FCz (*M* = 3.35 μV, *SE* = 0.56, *p* = .006) and Pz (*M* = 2.66 μV, *SE* = 0.57, *p* = .004). P3 amplitudes did not differ between FCz and Pz (*p* = 1). The feedback type × electrode location interaction approached significance, *F* (1, 42) = 2.45, *p* = .12, 𝜂_p_^2^ = .11. The difference between P3 amplitudes for wins and losses approached significance at electrode Pz (Win: *M* = 3.12 μV, *SE* = 0.67 & Loss: *M* = 2.20 μV, *SE* = 0.58, *p* = .11), but not at electrodes FCz (Win: *M* = 3.20 μV, *SE* = 0.61 & Loss: *M* = 3.50 μV, *SE* = 0.58, *p* = .47) and Cz (Win: *M* = 5.30 μV, *SE* = 0.58 & Loss: *M* = 5.10 μV, *SE* = 0.51, *p* = .61).

Collectively, the P3 results suggest that this component was not sensitive to outcome type in our non-monetary gambling task. Although there was a non-significant trend at electrode Pz for larger P3 amplitudes for wins relative to losses, this trend was not observed at electrodes FCz and Cz. As can be seen in the **Supplementary Figure**, there does not appear to be a RewP at electrode Pz. Thus, the sensitivity of the RewP (at electrodes FCz and Cz) to non-monetary outcomes in our data does not appear to be related to RewP and P3 component overlap.


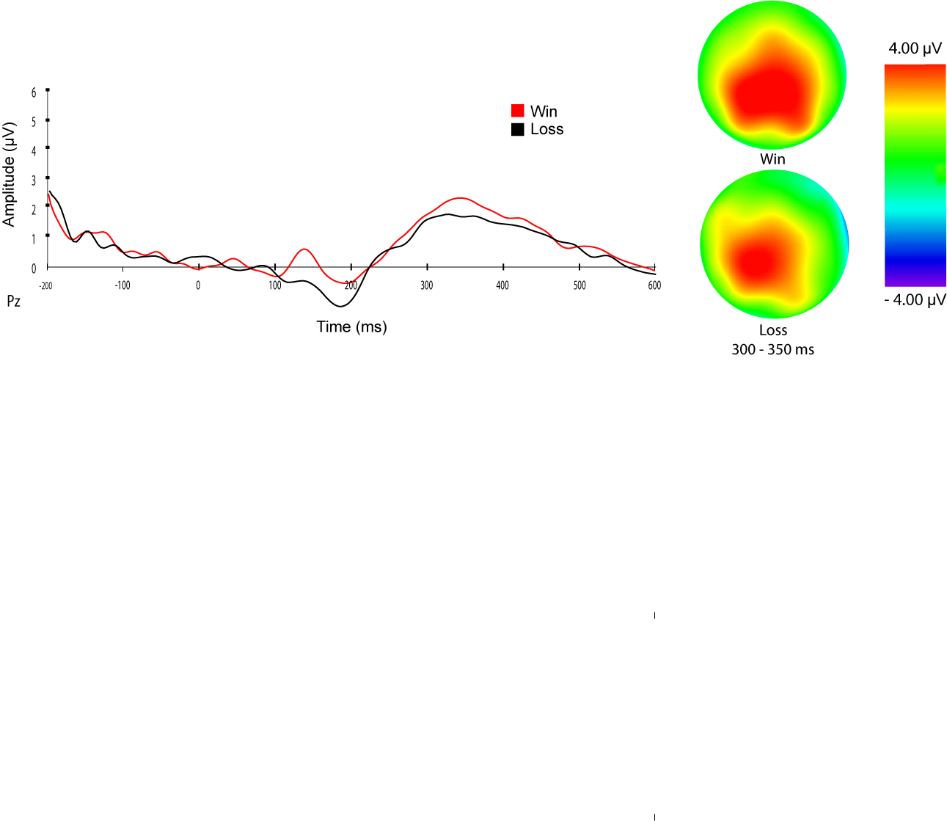


**Supplementary Figure.** *(Left)* *The event-related potential waveform at electrode Pz. P3 amplitudes for wins (red) were larger than losses (black), but this difference did not reach statistical significance. (Right) A map of electrocortical polarity at 300-350 ms after presentation of either win or loss feedback. Areas in red reflect maximal positive amplitudes.*
